# Erratum

**DOI:** 10.1002/ehf2.14108

**Published:** 2022-08-17

In the paper by Oh *et al*.,^1^ the authors noticed an error in Table 2, entry for ‘EF change’ under column ‘Later use (*n* = 48)’. The correct data is 13.4 ± 12.5 instead of 413.4 ± 12.5.

The publisher apologizes for this error.
